# Atypical findings of perineural cysts on postmyelographic computed tomography: a case report of intermittent intercostal neuralgia caused by thoracic perineural cysts

**DOI:** 10.1186/s12880-017-0210-z

**Published:** 2017-06-13

**Authors:** Hirokazu Iwamuro, Taro Yanagawa, Sachiko Takamizawa, Makoto Taniguchi

**Affiliations:** 1 Department of Neurosurgery, Tokyo Metropolitan Neurological Hospital, 2-6-1 Musashidai, Fuchu, 1830042 Japan; 2 Department of Research and Therapeutics for Movement Disorders, Juntendo University Graduate School of Medicine, 2-1-1 Hongo, Bunkyo-ku, Tokyo, 1138421 Japan

**Keywords:** Perineural cysts, Tarlov cysts, Computed tomography, Myelography, Intercostal nerves, Neuralgia, Thoracic wall, Nerve block

## Abstract

**Background:**

Perineural cysts are sometimes found incidentally with magnetic resonance imaging, and clinical symptoms requiring treatment are rare. Perineural cysts typically exhibit delayed filling with contrast medium on myelography, which is one of the criteria used by Tarlov to distinguish perineural cysts from meningeal diverticula. We present a case of multiple thoracolumbar perineural cysts, one of which was considered the cause of intermittent intercostal neuralgia with atypical findings on postmyelographic computed tomography seen as selective filling of contrast medium.

**Case presentation:**

A 61-year-old woman presented with intermittent pain on her left chest wall with distribution of the pain corresponding to the T10 dermatome. Magnetic resonance imaging showed multiple thoracolumbar perineural cysts with the largest located at the left T10 nerve root. On postmyelographic computed tomography immediately after contrast medium injection, the largest cyst and another at left T9 showed selective filling of contrast medium, suggesting that inflow of cerebrospinal fluid to the cyst exceeded outflow. Three hours after the injection, the intensity of the cysts was similar to the intensity of the thecal sac, and by the next day, contrast enhancement was undetectable. The patient was treated with an intercostal nerve block at T10, and the pain subsided. However, after 9 months of observation, the neuralgia recurred, and the nerve block was repeated with good effect. There was no recurrence 22 months after the last nerve block.

**Conclusions:**

We concluded that intermittent elevation of cerebrospinal fluid pressure in the cyst caused the neuralgia because of an imbalance between cerebrospinal fluid inflow and outflow, and repeated intercostal nerve blocks resolved the neuralgia. Our case demonstrates the mechanism of cyst expansion.

## Background

Perineural cysts, a subtype of meningeal cysts also called Tarlov cysts [1], are spinal extradural cysts at nerve roots. Most are asymptomatic, found incidentally with magnetic resonance imaging (MRI), and have an estimated prevalence between 1.2% [2] and 9.8% [3]. They commonly occur in the sacrum [4]. The etiology of these cysts is largely unknown; however, several studies have proposed that a ball-valve effect of cerebrospinal fluid (CSF) flow contributes to enlargement of the cysts [5], which is supported by delayed filling with contrast medium on myelography [6]. However, delayed filling implies only that CSF inflow into the cysts is restricted. We present a case of multiple thoracolumbar perineural cysts, some of which showed atypical findings on myelography as selective filling of contrast medium, suggesting restricted CSF outflow from the cysts.

## Case presentation

A 61-year-old woman presented with pain in her chest wall for two months, and had no significant medical history, such as trauma, infection, or tumor. The pain was located in the left lower chest in a band-like pattern overlapping the T10 dermatome. The pain was intermittent and occurred several times a day, continuing for 10–20 min each episode and alleviated when lying down. The pain was considered to be intercostal neuralgia.

**Fig. 1:**
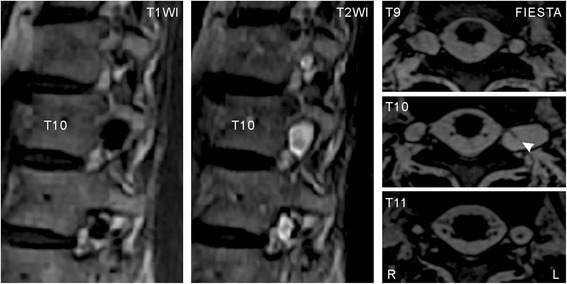
Magnetic resonance images at T9–T11 showing multiple perineural cysts. Sagittal views at the level of the left intervertebral foramina on T1- (*left*) and T2-weighted (*middle*) images show multiple cysts on the left side. In axial views acquired by fast imaging employing steady-state acquisition (FIESTA, *right*), a linear shadow (arrowhead) is seen in the largest cyst at left T10

**Fig. 2:**
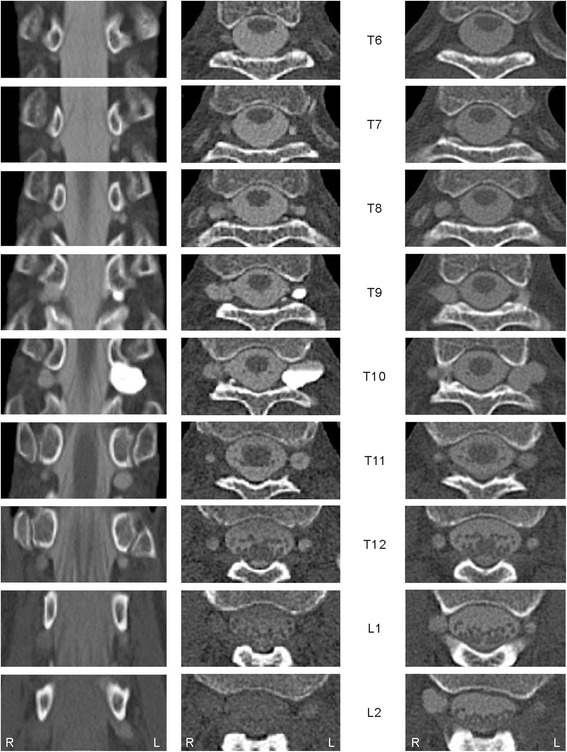
Computed tomography immediately after contrast medium injection (*left* and *middle* images) and 3 h later (*right* images). Two cysts at left T9 and T10 showed much higher intensity than the other cysts immediately after contrast medium injection

To identify the cause of the neuralgia, we performed MRI, which revealed multiple cysts at the T9–T11 nerve roots bilaterally (Fig. 1). No other causal abnormality for the chest wall pain was found other than the cysts. The interior of the cysts showed similar intensity to the CSF on imaging. The largest cyst was at the level of the left T10 vertebra and had a linear shadow that suggested the presence of nerve root fibers. Computed tomography (CT) with myelography was also performed to study CSF communication between the thecal sac and the cysts. After 10 mL of 240 mg I/mL iodinated contrast medium (Omnipaque 240, Daiichi Sankyo, Inc., Japan) was injected into the thecal sac at L4/5 in the lateral recumbent position, the first CT image was taken immediately (left and middle columns of Fig. 2), and revealed multiple cysts from T6–L2, including small cysts. The cysts were enhanced by the contrast medium and most showed the same intensity as that of the thecal sac at the corresponding level; however, the largest cyst at left T10 and another at left T9 showed much higher concentrations of the contrast medium. Three hours after the injection, the intensities of the cysts and the thecal sac were equal on the second CT (right column of Fig. 2). The next day, another CT examination showed no detectable contrast enhancement. Based on these findings, we suggested a diagnosis of multiple perineural cysts.

**Fig. 3:**
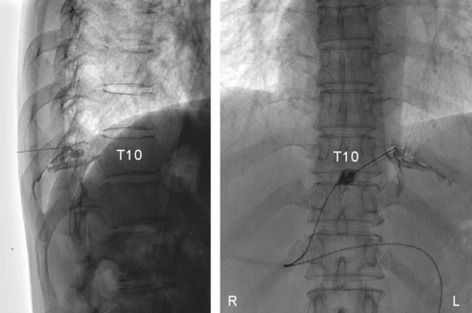
Thoracic X-ray images (*left*, lateral view; *right*, anterior-posterior view) during the left T10 intercostal nerve block. Contrast medium was injected to indicate the site of the nerve block

The patient was treated with an intercostal nerve block at T10 (Fig. 3) with 50 mg of mepivacaine hydrochloride (Carbocain, AstraZeneca, Inc., Japan) and 4 mg of dexamethasone sodium phosphate (DEXART, FujiPharma, Inc., Japan), and the pain subsided. Nine months later, the neuralgia recurred and another nerve block again relieved the pain. There was no recurrence 22 months after the last nerve block.

## Discussion

Perineural cysts are defined as CSF-filled saccular lesions in the extradural space of the spinal canal and are formed within the nerve root sheath at the dorsal root ganglion. Diagnosis should be based on histopathological findings because the presence of spinal nerve root fibers in the wall or cavity of the cyst are required for confirmation. However, magnetic resonance neurography has been recently proposed for diagnosis [7]. In our case, histopathology was not performed because non-surgical treatment was effective in relieving the associated neuralgia; but, results of the imaging studies supported our diagnosis.

Delayed filling with contrast medium on CT myelography is a typical finding with perineural cysts [6]. However, in our case, the largest cyst and one other showed immediate selective filling on the first CT, suggesting that the contrast medium flowed selectively into these cysts. We did not have an opportunity to observe the lesions directly, and therefore, do not know the exact mechanism of this inflow. However, considering the anatomical background of the lesions, we presume that the aspiration force was caused by negative pleural pressure. Several reports have stressed that delayed filling on CT myelography is a required finding for diagnosis of perineural cysts [6], but this may not always be true, especially in thoracic cases.

The etiology of perineural cysts remains unclear [8]. However, regarding cyst enlargement, the ball-valve theory has achieved consensus as an underlying mechanism, suggesting that enlargement is caused by restricted CSF outflow from the cyst as a result of CSF pulsatile and hydrostatic forces [9, 10]. The first CT results in our case suggested that CSF inflow into the two cysts exceeded outflow, which supports this theory.

We considered surgical treatment but ultimately decided to follow the patient conservatively because her symptoms resolved after the nerve block. It was difficult to prove a causal relationship between the perineural cysts and the intercostal neuralgia; however, we concluded that the chest wall pain was caused by the pressure of the largest cyst at left T10 for several reasons: 1) this cyst was considerably larger and involved the nerve root fibers that were causing the symptoms; 2) this cyst showed an imbalance between CSF inflow and outflow; 3) because the pain was intermittent and alleviated when the patient was lying down, the cause of the pain was expected to be easily resolved, considering its relationship to head position; and 4) we found no other causal abnormality to explain the neuralgia other than the cyst. If the patient’s symptoms reappear in the future, we would reconsider invasive treatment as the patient would meet the criteria for surgery [9].

## Conclusions

We report a rare case of thoracolumbar multiple perineural cysts, one of which was considered the cause of intermittent intercostal neuralgia. The cyst showed immediate selective filling of the contrast medium on CT myelography, which suggested an aspirating force resulting from pleural negative pressure. Because repeat intercostal nerve blocks resolved the neuralgia and we did not perform surgery, our suggested etiology was difficult to prove directly; however, we concluded that intermittent elevation of pressure in the cyst caused the neuralgia because of an imbalance between CSF inflow and outflow. This case demonstrates the mechanism of perineural cyst expansion.

## Abbreviations

CSF: Cerebrospinal fluid
CT: Computed tomography
FIESTA: Fast imaging employing steady-state acquisition
MRI: Magnetic resonance imaging.
